# Dysfunctional Personality Beliefs Linked to Emotion Recognition Deficits in Individuals With Cocaine Addiction and Personality Disorders

**DOI:** 10.3389/fpsyt.2019.00431

**Published:** 2019-06-18

**Authors:** Natalia Albein-Urios, Jose M. Martinez-Gonzalez, Oscar Lozano-Rojas, Antonio Verdejo-Garcia

**Affiliations:** ^1^Cognitive Neuroscience Unit, School of Psychology, Deakin University, Geelong, VIC, Australia; ^2^Centro Provincial de Drogodependencias, Diputación de Granada, Granada, Spain; ^3^Departamento de Psicología Clínica, Experimental y Social, Universidad de Huelva, Huelva, Spain; ^4^Research Center on Natural Resources, Health and Environment, University of Huelva, Huelva, Spain; ^5^Turner Institute for Brain and Mental Health, Monash University, Melbourne, VIC, Australia

**Keywords:** emotion recognition, personality disorders, cocaine use disorder, personality beliefs, antisocial beliefs

## Abstract

**Background:** Facial emotion recognition is impaired in addiction and personality disorders. Dysfunctional personality beliefs reflect negative interpersonal schemas that may underpin emotion recognition deficits. We aimed to examine the association between personality beliefs and emotion recognition among participants with cocaine use disorder including those with comorbid personality disorders.

**Methods:** We recruited 70 participants with cocaine use disorder aged between 19 and 52 who had used 14 g of cocaine over 4.8 years on average. Thirty-eight participants had an additional personality disorder (11 Borderline, 7 Histrionic, 5 Antisocial, 10 Avoidant, and 5 Obsessive–Compulsive). Dysfunctional beliefs were indicated with the Personality Belief Questionnaire, and facial emotion recognition was indicated with the Ekman’s Test. We applied correlations/multiple regressions to test the relationship between beliefs and emotion recognition.

**Results:** Personality beliefs reflecting paranoid, borderline, and antisocial schemas were negatively associated with emotion recognition. Antisocial beliefs were associated with poorer recognition of fear, and paranoid beliefs with poorer recognition of disgust. Antisocial beliefs were significantly associated with emotion recognition after adjusting for cocaine use.

**Conclusion:** Dysfunctional personality beliefs are associated with poorer emotion recognition in cocaine addiction. Personality-related negative schemas about the self and others can impact social cognition and interaction during cocaine treatment.

## Introduction

Facial emotion recognition reflects the ability to identify basic emotions in others and is essential for adaptive social interaction (1, 2). Deficits in facial emotion recognition are a hallmark of substance use disorders (SUD) (3). However, although SUD often co-occur with personality disorders (4), little is known on the relationship between comorbid personality dysfunction and facial emotion recognition. This is important because personality disorders are characterized by difficulties with social interaction and disturbed representations of others (5–7). Individuals with personality disorders have lower facial emotion recognition accuracy than healthy controls (8–10). However, we do not know if the comorbidity between SUD and personality disorders is associated with additive or specific impacts on facial emotion recognition. Unraveling the link between personality dysfunction and facial emotion recognition can contribute to understand social interaction problems and persistence of SUD among individuals with comorbid personality disorders.

In the context of SUD without comorbidities, previous studies have found that individuals with cocaine-SUD have poorer recognition of specific emotions such as anger and fear (11). In the only study comparing individuals with cocaine-SUD with and without personality disorders, Morgan and Marshall (12) found no significant effects of comorbidity on fear recognition. Using psychophysiological measures of fear and arousal reactivity, Baschnagel et al. (13) also failed to find a significant effect of the comorbidity on psychophysiological measures of emotion processing. However, these studies have adopted a categorical approach, by comparing comorbid versus non-comorbid participants (13) or covarying the effect of the comorbid personality diagnosis (12). However, current evidence supports the view that dimensional measures of personality dysfunction are better suited than categorical approaches to gain insight on emotion recognition deficits (14). Dimensional measures of antisociality and anxiety are negatively associated with emotion recognition accuracy, and specifically with poorer recognition of anger and fear among healthy individuals (15).

Personality beliefs are key dimensional features of personality disorders that have been neglected in previous studies of emotion recognition (16, 17). Dysfunctional beliefs reflect deep-rooted negative schemas that can consistently bias cognitive and affective judgments about oneself and others (17). Since these negative schemas are linked to specific personality disorders, the degree of disturbance can be estimated by measuring endorsement of specific sets of beliefs (e.g., antisocial—“I should do whatever I can get away with”; obsessive–compulsive—”Any flaw or defect may lead to a catastrophe”) (18). The Personality Belief Questionnaire (PBQ) was originally designed to measure these personality beliefs and has received recent attention and excellent kudos as a dimensional measure of personality dysfunction that is well aligned with contemporary views, as well as reliable and predictive of severity of personality dysfunction (17, 19). Previous studies have shown that individuals with cocaine-SUD have elevated PBQ scores compared to healthy controls (20, 21). Moreover, those with cocaine-SUD and personality disorders exhibit higher scores than controls in antisocial, borderline, histrionic, and narcissistic scales (20). PBQ scores are also elevated among people with other psychiatric disorders (i.e., depression and eating disorders) who have comorbid personality disorders compared to those with single diagnoses (17, 19).

In this study, we aimed to examine the relationship between dimensional estimates of dysfunctional personality beliefs, measured with the PBQ, and emotion recognition, indicated by the gold-standard Ekman facial emotion recognition test, among people with cocaine-SUD including those with comorbid personality disorders. In fitting with previous evidence on dimensional personality correlates of emotion recognition, we hypothesized that dysfunctional beliefs associated with antisocial and anxious-like personality disorders would be linked to lower emotion recognition accuracy and specifically poorer recognition of fear and anger.

## Methods

### Participants

The sample comprised 70 participants (11 females) with cocaine use disorder, of whom 38 (54%; consistent with previously reported comorbidity rates) (22) had comorbid personality disorders (11 Borderline, 7 Histrionic, 5 Antisocial, 10 Avoidant, and 5 Obsessive–Compulsive). Participants with and without comorbid personality disorders did not significantly differ on sociodemographic characteristics or cocaine use patterns (**Table 1**). All participants were recruited from a city-wide public outpatient addiction treatment center in Granada (Spain). Treatment consisted of cognitive behavioral therapy and psychosocial support. The inclusion criteria were as follows: i) meeting Diagnostic and Statistical Manual of Mental Disorders, Fourth Edition, Text Revision (DSM-IV-TR) (23) criteria for cocaine dependence indicated with the Structured Clinical Interview for DSM-IV Disorders–Clinical Version (SCID-CV) (24), ii) being abstinent for at least 2 weeks indicated by self-report and regular urine analyses, and iii) IQ levels ≥80 (to ensure facial emotion recognition was not impacted by general cognitive dysfunction) indicated with the Kaufman Brief Intelligence Test (25). Personality disorders included in the DSM-IV-TR were diagnosed by an accredited clinical psychologist (JM-G) using the International Personality Disorders Examination (26). Participants received personality disorders diagnosis at the same time as cocaine dependence diagnosis. The exclusion criteria were as follows: i) other Axis I comorbid disorders, with the exceptions of alcohol abuse and nicotine dependence, indicated with the SCID-CV; ii) history of head injury and/or neurological, infectious, systemic, or any other diseases affecting the central nervous system, indicated by self-report and clinical records.

**Table 1:** Sociodemographic characteristics, drug use patterns, and personality beliefs and emotion recognition scores in participants with and without personality disorders.

	Whole sample (*n* = 70)	SUD (*n* = 32)	SUD + PD (*n* = 38)	*t*	*p*
Age	33.53 (6.84)	32.60 (6.38)	34.32 (7.19)	−1.050	0.297
Education (yrs.)	10.20 (1.77)	10.00 (1.61)	10.37 (1.91)	−0.864	0.391
Cocaine grams/mo.	14.00 (19.92)	14.45 (20.15)	13.63 (20.01)	0.169	0.866
Cocaine duration (mo.)	57.37 (51.32)	55.02 (53.10)	59.29 (50.45)	−0.342	0.734
PBQ Paranoid	19.91 (13.63)	16.56 (12.68)	22.73 (13.92)	−1.925	0.058
PBQ Schizoid	23.04 (10.96)	21.22 (10.45)	24.58 (11.29)	−1.283	0.204
PBQ Antisocial	17.21 (8.51)	15.84 (8.13)	18.37 (8.75)	−1.242	0.219
PBQ Borderline	16.41 (10.29)	12.28 (9.48)	19.89 (9.75)	−3.296	0.002*
PBQ Histrionic	16.66 (8.58)	15.09 (7.18)	17.97 (9.51)	−1.408	0.164
PBQ Narcissistic	12.04 (7.65)	11.75 (8.80)	12.29 (6.62)	−0.294	0.769
PBQ Avoidant	17.44 (9.06)	14.44 (8.89)	19.97 (8.51)	−2.657	0.010
PBQ Dependent	20.26 (10.52)	17.41 (8.94)	22.73 (11.25)	−2.152	0.035
PBQ O–C	25.16 (10.78)	23.41 (11.57)	26.63 (9.98)	−1.252	0.215
Total Emotion Recognition	48.74 (4.94)	49.40 (4.41)	48.18 (5.34)	1.032	0.306

SUD, substance use disorder (cocaine); PD, personality disorder; yrs., years; mo., months; PBQ, Personality Beliefs Questionnaire; O–C, Obsessive–compulsive. *p < 0.005.

### Measures

*Interview for Research on Addictive Behavior* (27): This semi-structured interview collects information about substance use patterns (i.e., dosage, frequency, and duration) and yields two main measures: monthly use of each substance (quantity per month) and total duration of use of each substance (duration in months).

*Personality Belief Questionnaire (PBQ)* (18): The PBQ was administered to dimensionally measure dysfunctional beliefs or negative schemas associated with personality disorders. It is a 126-item self-report questionnaire that measures the degree of endorsement of dysfunctional beliefs associated with personality disorders, i.e., paranoid, schizoid, antisocial, borderline, histrionic, narcissistic, avoidant, dependent, and obsessive–compulsive beliefs. The Spanish version of the scale that we used in this study has demonstrated sound psychometric characteristics (28).

*Ekman Faces Test (EFT)*: This is a computer task that assesses recognition of facial emotional expressions. The task uses stimuli from the Facial Expressions of Emotion: Stimuli and Tests (FEEST) (29). We presented 60 faces depicting expressions of anger, disgust, fear, happiness, sadness, and surprise (6 emotions, 10 faces each). Each face was presented on a computer monitor for a maximum of 5 s, after which individuals were asked to select the emotion that best described it. The performance measure was the sum score of total correct identifications (total recognition: range, 0–60).

### Procedures

The Human Research Ethics Committee of the University of Granada approved the study. All participants provided written informed consent. Participants underwent two assessment sessions: one to diagnose substance use and personality disorders, and a second one to complete personality beliefs and emotion recognition measures, along with other cognitive measures that have been published elsewhere.

### Analyses

First, we contrasted emotion recognition scores between participants with and without personality disorders using *t*-tests. Next, we examined the relationship between personality beliefs and total emotion recognition scores using *Spearman* correlation coefficients. When there was a significant association between specific dysfunctional beliefs and total emotion recognition, we run additional correlations between such beliefs and discrete emotions recognition scores (e.g., anger and fear). Finally, we tested if the relationship between dysfunctional beliefs and total emotion recognition scores stood after adjusting for sociodemographic characteristics and lifetime substance use using multiple regression. Results from group contrasts and correlational analyses, involving multiple tests, were considered significant if *p* values were below 0.005 to protect against Type I error. Results from targeted regression analyses were considered significant at the standard *p* < 0.05 value. Data is available at https://monash.figshare.com/s/f35e993c96fbb2899ecb.

## Results

### Emotion Recognition in Participants With Versus Without Personality Disorders

We found no significant differences between participants with and without personality disorders in total emotion recognition scores (**Table 1**). As expected, participants with personality disorders had generally higher PBQ scores (reflecting greater endorsement of dysfunctional personality beliefs), but the group differences were only significant for borderline beliefs (**Table 1**).

### Relationship Between Emotion Recognition and Dysfunctional Personality Beliefs

We found significant negative associations between the total emotion recognition score and antisocial, borderline, and paranoid beliefs (**Table 2**). Subsequent analyses showed that antisocial beliefs were negatively associated with recognition of fear, *r* = −0.376, *p* = 0.001, whereas paranoid beliefs were negatively associated with recognition of disgust, *r* = −0.372, *p* = 0.002 (**Supplementary Table S1**).

**Table 2:** Correlations between dysfunctional personality beliefs and emotion recognition.

	Emotion recognition total score	
	*rho*	*p*
PBQ Paranoid	−0.359	0.002*
PBQ Schizoid	−0.186	0.122
PBQ Antisocial	−0.399	0.001*
PBQ Borderline	−0.355	0.003*
PBQ Histrionic	−0.133	0.272
PBQ Narcissistic	−0.212	0.080
PBQ Avoidant	−0.247	0.039
PBQ Dependent	−0.321	0.007
PBQ Obsessive–Compulsive	−0.329	0.005

PBQ, Personality Beliefs Questionnaire. *p < 0.005.

### Regression Analyses Adjusted by Sociodemographic and Drug Use Characteristics

After adjusting for age, education, and lifetime drug use, antisocial beliefs were significantly associated with total emotion recognition scores (*F*_full model_ = 3.647, *Adj R^2^*_full model_ = 0.214, *p*_full model_ = 0.002, *Beta*_antisocial_ = −0.342, *p*_antisocial_ = 0.019) (**Table 3**). No other individual predictors were significantly associated with emotion recognition.

**Table 3:** Multiple regression model entering sociodemographic characteristics, drug use patterns, and dysfunctional beliefs as predictors of emotion recognition.

	Age		Education		Cocaine (gr)		Cocaine (mo)		Paranoid		Antisocial		Borderline	
	*β*	*p*	*β*	*p*	*β*	*p*	*β*	*p*	*β*	*p*	*β*	*p*	*β*	*p*
Emotion recognition total score	−0.031	0.813	0.139	0.253	−0.021	0.860	−0.070	0.586	0.144	0.482	−0.342	0.019*	−0.322	0.109

gr, grams; mo, months; O-C. *p < 0.05.

## Discussion

Our findings show that, although participants *with* and *without* personality disorders did not differ in emotion recognition, the degree of endorsement of dysfunctional personality beliefs was negatively associated with facial emotion recognition accuracy. These results suggest that individuals with more negative schemas associated with personality dysfunction can have greater problems to identify and interpret emotions in others, and ultimately more social interaction problems.

The link between dysfunctional personality beliefs and poorer emotion recognition provides support to the notion that maladaptive personality schemas are associated with social interaction deficits in people with SUD (30, 31). This relationship is acknowledged in modern definitions of personality disorders and stimulant addiction, which refer to disturbances in interpersonal functioning (32, 33). The directionality of the association is unclear. It is possible that emotion recognition deficits predate personality dysfunction and thus contributes to the formation of dysfunctional beliefs *via* early negative social interaction experiences (34). It is also plausible that dysfunctional beliefs cause stable biases in affective judgment that ultimately impact emotion recognition (e.g., “Others will try to use me or manipulate me if I don’t watch out”) (16). Since participants were in the “craving phase” of their SUD (35), it is also possible that state-related symptoms such as anhedonia modulate the link between personality and emotion recognition (36). Furthermore, the link between emotion recognition and dysfunctional personality beliefs, which are dimensional measures of personality dysfunction, supports the view that dimensional (versus categorical) indices of personality dysfunction can be more tightly aligned with social cognition and interaction phenotypes (37). Although emotion recognition is a well-recognized index of social cognition skills (1, 2), our findings can also stimulate further research on other aspects of social cognition and interaction in the context of addiction and personality disorders.

The link between specific personality beliefs and difficulties to recognize emotions in others has also important clinical value. In fact, we found specific associations between antisocial beliefs and poorer recognition of fear, which is consistent with previous findings among individuals with antisocial personality disorder (38) and align with the “low fear” theory of antisocial personality and psychopathy (39). Since fear recognition is essential to avoid risk (e.g., by recognizing others’ appraisal about potentially risky situations such as those conducive to relapse) and harm to others (e.g., by recognizing their fear in response to one’s actions), individuals with greater endorsement of antisocial beliefs and poorer emotion recognition might be at particularly high risk of poor clinical outcomes (40). We also found negative associations between paranoid beliefs and poorer recognition of disgust, but this relationship did not survive adjustment for sociodemographic and clinical characteristics. Therefore, these relationships might be conflated with other indicators of severity (e.g., higher levels of drug use) and should be reassessed in future studies. Establishing these links is important, since little is known about the social cognition correlates of personality dysfunctions associated with paranoid schemas compared to antisocial or borderline schemas (38).

Our findings need to be appraised in the context of relevant limitations. First, results are cross-sectional and correlational, meaning that we cannot draw causal conclusions. Second, we focused on two very specific indices of personality dysfunction (beliefs) and social cognition (emotion recognition), and hence, more comprehensive assessments are needed to confirm if the relationship between these constructs stands in the context of other indices of personality dysfunction (e.g., dimensional diagnostic tools) and social cognition (e.g., empathy). Third, according to the cognitive theory of personality disorders (41), participants with personality disorders should have generally elevated dysfunctional beliefs; the fact that we only found differences in borderline beliefs may be due to the small number of cases. Fourth, although we interpret findings mostly in the context of personality dysfunction, other etiological and clinical aspects of cocaine addiction (e.g., genetic vulnerability and cocaine dosage) may also contribute to emotion recognition deficits.

## Ethics Statement

The Human Research Ethics Committee of the University of Granada (Spain) approved this study.

## Author Contributions

AV-G, JM-G and OL-R designed the study. NA-U and JM-G conducted assessments. OL-R and AV-G conducted statistical analyses. NA-U and AV-G wrote a first draft of the manuscript, which was reviewed by all authors.

## Funding

This study was funded by project grant COPERNICO (2009/052) from the Spanish Ministry of Health. Funding for open access is provided by internal funds of Monash University and Deakin University.

## Conflict of Interest Statement

The authors declare that the research was conducted in the absence of any commercial or financial relationships that could be construed as a potential conflict of interest.

The handling editor is currently co-organizing a Research Topic with one of the authors AV, and confirms the absence of any other collaboration.
