# Supplementary material for: Dysfunctional Personality Beliefs Linked to Emotion Recognition Deficits in Individuals With Cocaine Addiction and Personality Disorders
Source: Front Psychiatry. 2019 Jun 18;10:431. doi: 10.3389/fpsyt.2019.00431 (PMC6591705; doi:10.3389/fpsyt.2019.00431)
Supplement: Supplementary file 1 [file Table_1.docx]

|  |  | Anger | Disgust | Fear | Happiness | Sadness | Surprise |
| --- | --- | --- | --- | --- | --- | --- | --- |
| Paranoid | *Rho* | -0.010 | -0.372 | -0.209 | -0.189 | -0.157 | -0.181 |
|  | *p* | 0.933 | 0.002* | 0.083 | 0.188 | 0.195 | 0.133 |
| Antisocial | *Rho* | -0.083 | -0.250 | -0.376 | -0.295 | -0.201 | -0.064 |
|  | *p* | 0.496 | 0.037 | 0.001* | 0.013 | 0.096 | 0.598 |
| Borderline | *Rho* | -0.161 | -0.328 | -0.184 | -0.186 | -0.221 | -0.061 |
|  | *p* | 0.182 | 0.006 | 0.127 | 0.122 | 0.066 | 0.617 |

**Supplementary Table S1.** Spearman correlations between personality beliefs and recognition of specific emotions

Note. *p<0.005
